# Characterization of *Brachypodium distachyon* as a nonhost model against switchgrass rust pathogen *Puccinia emaculata*

**DOI:** 10.1186/s12870-015-0502-9

**Published:** 2015-05-08

**Authors:** Upinder S Gill, Srinivasa R Uppalapati, Jin Nakashima, Kirankumar S Mysore

**Affiliations:** Plant Biology Division, The Samuel Roberts Noble Foundation, Ardmore, Oklahoma 73401 USA; Current address: Biologicals and Fungicide Discovery, DuPont Crop Protection, Newark, DE 19711 USA

**Keywords:** *Brachypodium*, Switchgrass, *Puccinia emaculata*, Nonhost resistance

## Abstract

**Background:**

Switchgrass rust, caused by *Puccinia emaculata*, is an important disease of switchgrass, a potential biofuel crop in the United States. In severe cases, switchgrass rust has the potential to significantly affect biomass yield. In an effort to identify novel sources of resistance against switchgrass rust, we explored nonhost resistance against *P. emaculata* by characterizing its interactions with six monocot nonhost plant species. We also studied the genetic variations for resistance among *Brachypodium* inbred accessions and the involvement of various defense pathways in nonhost resistance of *Brachypodium*.

**Results:**

We characterized *P. emaculata* interactions with six monocot nonhost species and identified *Brachypodium distachyon* (Bd21) as a suitable nonhost model to study switchgrass rust. Interestingly, screening of *Brachypodium* accessions identified natural variations in resistance to switchgrass rust. *Brachypodium* inbred accessions Bd3-1 and Bd30-1 were identified as most and least resistant to switchgrass rust, respectively, when compared to tested accessions. Transcript profiling of defense-related genes indicated that the genes which were induced in Bd21after *P. emaculata* inoculation also had higher basal transcript abundance in Bd3-1 when compared to Bd30-1 and Bd21 indicating their potential involvement in nonhost resistance against switchgrass rust.

**Conclusion:**

In the present study, we identified *Brachypodium* as a suitable nonhost model to study switchgrass rust which exhibit type I nonhost resistance. Variations in resistance response were also observed among tested *Brachypodium* accessions. *Brachypodium* nonhost resistance against *P. emaculata* may involve various defense pathways as indicated by transcript profiling of defense related genes. Overall, this study provides a new avenue to utilize novel sources of nonhost resistance in *Brachypodium* against switchgrass rust.

**Electronic supplementary material:**

The online version of this article (doi:10.1186/s12870-015-0502-9) contains supplementary material, which is available to authorized users.

## Background

Switchgrass (*Panicum virgatum* L.) is considered a potential biofuel crop by the United States Department of Energy (DOE) [1]. Switchgrass can grow on marginal lands with low-input agriculture and without many crop management practices. Due to extensive root systems and clumping growth patterns, switchgrass can provide protection against soil erosion and also acts as an excellent habitat for wildlife [2]. Since switchgrass is perennial and a monoculture crop, it can become more susceptible to pathogens and insects. However, to date, very limited information is available on diseases of switchgrass [3]. Among diseases of switchgrass, switchgrass rust, caused by *Puccinia emaculata*, is economically very important since it has the potential to significantly affect biomass yield. *P. emaculata* is a biotrophic fungal pathogen and is widely distributed in switchgrass growing regions of North America with a moderate to high incidence of infection [4-9]. Genetic variations for rust resistance exist in natural populations of switchgrass and have been studied in detail [6,8]. In general, lowland switchgrass cultivars such as Alamo and Kanlow are moderately resistant to *P. emaculata* compared to upland cultivars such as Summer and Cave-in-Rock [8]. Variations also exist in the virulence of urediniospores collected from different sources [10]. Urediniospores of *P. emaculata* collected from ornamental switchgrass were found to have greater virulence than urediniospores collected from agronomic switchgrass plots [10]. These variations in virulence of wind-borne rust urediniospores pose a great threat to monoculture of switchgrass varieties in new geographical areas. Genetic variations in switchgrass germplasm can be exploited to find sources of host resistance, but host resistance is generally less durable due to the fact that variations also exist in rust pathogen isolates.

Nonhost resistance (NHR), on the other hand, is a form of durable resistance shown by all members of a plant species against all isolates of a specific pathogen [11]. NHR response in the plant may not lead to any visual symptoms (type I), or it can be associated with visible symptoms (type II), depending on the host-pathogen interaction [12]. During nonhost interactions, the first line of defense involves preformed physical and chemical barriers such as surface topology, cytoskeleton, antimicrobial compounds and secondary metabolites [12-14]. The importance of wax layers on leaf surfaces has been described specifically for NHR against fungal pathogens where epicuticular wax affects fungal pre-infection structures [15,16]. An inducible defense response is often triggered if the primary line of defense is breached by the pathogen [14]. Generally, conserved elicitor molecules, often called microbe- or pathogen-associated molecular patterns (MAMPS or PAMPs), are sensed by plant plasma membrane receptors to trigger basal or NHR response [17]. In certain situations, if a nonhost species is closely related to a host species for a particular pathogen, the NHR response is often associated with hypersensitive (HR) response [18,19].

The study of NHR against biotrophic rust pathogens, which usually infect via urediniospores and pass through a set of defined developmental stages, is potentially more informative because resistance at each stage of development can be precisely defined [20]. NHR against rusts typically happens before haustoria formation during pre-penetration events or due to restrictive fungal growth in the substomatal cavity [21]. In some cases, post-haustorial resistance of rust fungi is also observed [22]. Rust diseases of cereals and other grasses are mainly caused by rust fungi belonging to the genus *Puccinia*, and it is considered the most economically destructive genus of biotrophic fungi [23]. NHR mechanisms against rust fungi of wheat have been studied using divergent species such as Arabidopsis and broad bean (*Vicia faba* L.), and also closely related species such as *Brachypodium distachyon*, barley and rice [20,22,24-26]. Among these species, rice is the only monocot known so far which is immune to all rust pathogens and shows an active NHR response against cereal rust pathogens by involving hydrogen peroxide production and callose depositions [24]. Quantitative trait loci (QTL) analysis of NHR in barley against *P. triticina* (wheat leaf rust) identified map locations similar to genes conferring partial resistance to *P. hordei*, a pathogen of barley [26].

*Brachypodium*, which is considered a model plant species for the study of some members of the family *Poaceae*, is a host to the rust pathogen *P. brachypodii*. Variations exist among *Brachypodium* accessions for resistance against *P. brachypodii*, and QTLs for resistance have been identified [27,28]. Since its acceptance of *Brachypodium* as a model species by the scientific community at the start of the 21^st^ century, a variety of genetic and genomic resources have been developed, such as T-DNA insertion lines, efficient genetic transformation and sequencing of the whole genome [29-31]. *Brachypodium* has been used as a nonhost to study plant diseases caused by a variety of plant pathogens including cereal rusts, *Fusarium* head blight of wheat, rice blast, powdery mildew and *Barley stripe mosaic virus* (BSMV) [24,32-36]. *Brachypodium* accessions show large variations in NHR response against cereal rust pathogens [33]. In some instances, sporulating pustules of *P. striiformis* (a wheat pathogen) appeared on a few of the tested *Brachypodium* accessions [33]. Variations in resistance were also reported in *Brachypodium* against *P. graminis* f. sp. *tritici*, *lolii* and *phlei-pratensis*, where many of the tested accessions showed sporulating pustules against *P. graminis* f. sp. *lolii* and *phlei-pratensis* [34]. Using genetic mapping populations of *Brachypodium* ecotypes, the inheritance of variations against *P. striiformis* f. sp. *tritici* were studied [24]. Genetic analysis indicated a relatively simple inheritance of NHR in *Brachypodium*, including single gene segregation in one of the families [24].

Here we present our results on identification of *Brachypodium* as a suitable plant to study nonhost resistance against *P. emaculata* and detailed characterization of *Brachypodium-P. emaculata* nonhost interactions involving *Brachypodium* inbred accessions. The transcript level of plant defense-related genes was also studied to understand genetic variation for resistance among these accessions.

## Results

### Identification of an appropriate nonhost monocot plant species to study NHR against *P. emaculata*

**Figure 1:**
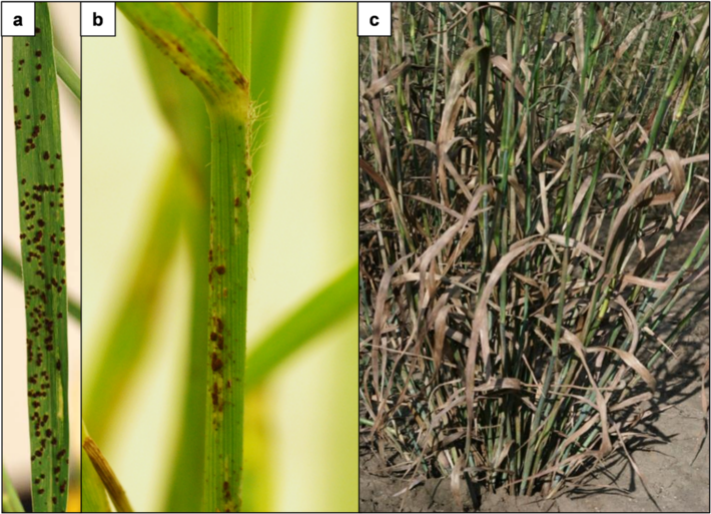
Switchgrass cv. Summer infected with switchgrass rust. Switchgrass cv. Summer plants were inoculated by spraying *P. emaculata* urediniospores at a concentration of 10^5^ spores per milliliter under controlled conditions. Two weeks after inoculation, dark brown rust pustules containing dikaryotic urediniospores appeared on leaf **(a)** and stem **(b)** of switchgrass plants. **(c)** A switchgrass plant in a field severely infested with rust under natural conditions.

**Figure 2:**
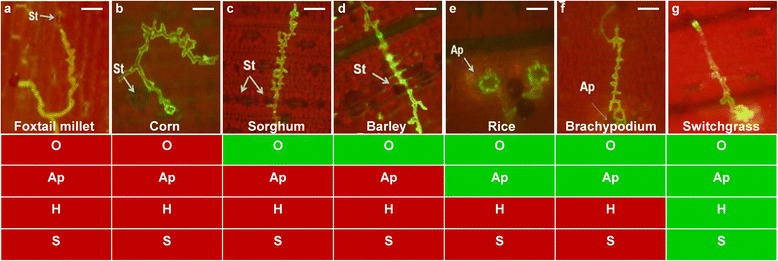
*P. emaculata* urediniospore interactions with nonhost and host monocot plant species. Detached leaves of each species were arranged in Petri plates and spray-inoculated with *P. emaculata* urediniospores. After 72 h, leaves were immersed in 1x PBS containing 10 μg/ml of WGA-Alexa-fluor-488. Epifluorescence micrograph visualized under an epifluorescence microscope showed pre-infection structures of *P. emaculata* on foxtail millet **(a)**, corn **(b)**, sorghum **(c)**, barley **(d)**, rice **(e)**, *Brachypodium* (Bd21) **(f)** and switchgrass **(g)**. Germinated urediniospores on the leaf surface were evaluated for oriented growth (O), appressoria formation (Ap) on stomata (St), haustoria formation (H) and sporulating rust pustules (S). Boxes below each figure represent absence (red) and presence (green) of studied pre-infection structures of *P. emaculata*; scale bar = 50 μm.

To identify a suitable monocot nonhost model system for *P. emaculata,* we screened several monocot plants from BEP (Bambusoideae, Ehrhartoideae and Pooideae) and PACCMAD (Panicoideae, Arundinoideae, Chloridoideae, Centothe-coideae, Micrairoideae, Aristidoideae and Danthonioideae) clades (Additional file 5). As reported previously, switchgrass cv. Summer is a susceptible host to *P. emaculata* [8]. As expected, switchgrass cv. Summer was infected with rust urediniospores and showed disease symptoms in the form of sporulating pustules under controlled (Figure 1a, b) and natural conditions (Figure 1c). *P. emaculata* germ tubes failed to recognize the host surface, followed by a lack of oriented growth of germ tube and appressoria formation on the abaxial or adaxial leaf surfaces of corn, sorghum or foxtail millet belonging to the PACCMAD clade (Figure 2a, b, c). However, on sorghum leaf surfaces, few urediniospore germ tubes were able to show oriented growth similar to that of the host plant, switchgrass (Figure 2c). Oriented growth of rust spore germ tubes was also noticed on the leaf surfaces of both barley and rice, but the appressoria were developed only in the case of rice (Figure 2d, e).

**Figure 3:**
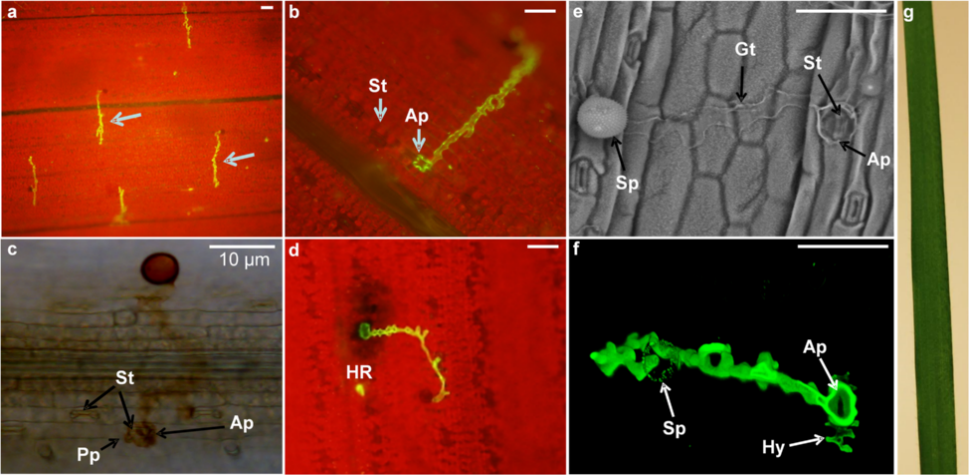
*P. emaculata* urediniospore interactions with *Brachypodium*. Epifluorescence micrograph showing pre-infection structures after germination of *P. emaculata* urediniospores (Sp) on *Brachypodium* (Bd21) leaf surface in the form of the oriented growth pattern of germ tubes (Gt) **(a)** and formation of appressoria (Ap) on stomatal (St) openings **(b)**. **(c)** 3, 3’-diaminobenzidine (DAB)-stained fungal structures showing a penetration peg (Pp) originating from the appressorium that helps to push through the closed stomata for entry into the intercellular space within the host leaf. **(d)** Localized cell death in the form of hypersensitive (HR) response at the site of infection. **(e)** Scanning electron microscope (SEM) image showing the formation of pre-infection structures after urediniospore germination on the leaf surface of *Brachypodium*. **(f)** Confocal microscope image showing intercellular hyphal (Hy) growth inside mesophyll cells. **(g)** *Brachypodium* leaf at two weeks after infection with rust urediniospores. No visible disease symptoms appeared on *Brachypodium*. Scale bar = 50 μm or unless specified.

On *Brachypodium* accession Bd21, a commonly used accession for which the genome sequence is available [31] and belonging to the BEP clade, *P. emaculata* germ tubes showed oriented growth perpendicular to the long axis of the epidermal cells (Figures 2f and 3a). The oriented growth of germ tubes indicates recognition of topographic and chemical signals on the host surface. Furthermore, the germ tubes encountered stomata and formed appressoria on *Brachypodium* (Figure 3b, e). Further infection occurred by formation of a penetration peg which is presumably followed by fungal hyphae growth in mesophyll cells (Figure 3c, f). In some cases, microscopic evaluation also revealed hypersensitive cell death at the site of fungal penetration (Figure 3d). However, disease symptoms in the form of urediniospore-containing rust pustules or hypersensitive resistance response were not visually observed on *Brachypodium* leaves (Figure 3g) thus representing type I NHR as proposed previously [12]. These results suggest that *Brachypodium* would be a suitable model plant among the monocot plants tested to identify signaling components to NHR against *P. emaculata*.

### Variability of *Brachypodium* inbred accessions for resistance against *P. emaculata*

**Figure 4:**
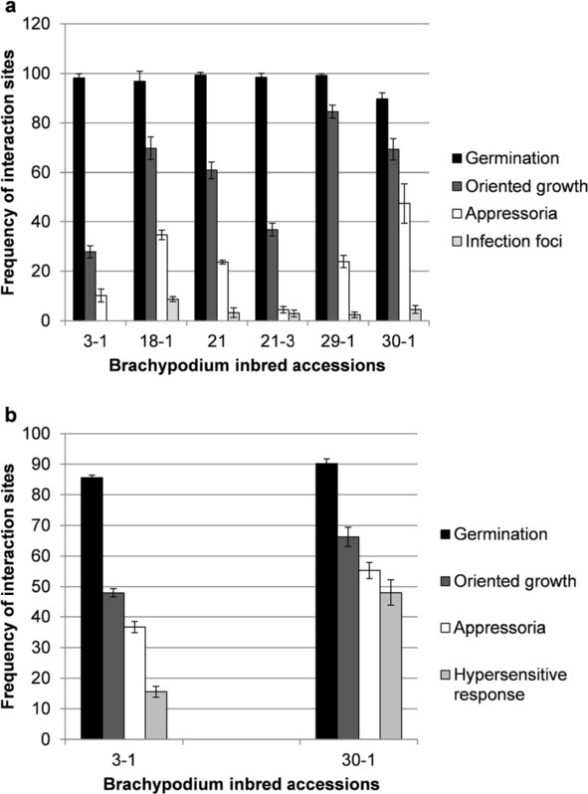
Genetic variations for rust pre-infection structures on *Brachypodium* inbred accessions. Detached leaves of accessions Bd3-1, Bd18-1, Bd21, Bd21-3, Bd29-1 and Bd30-1 were spray-inoculated with *P. emaculata* urediniospores followed by staining with WGA-Alexa-fluor-488, 72 h after inoculation. Stained fungal structures were visualized and quantified by epifluorescence microscope. Percent of *P. emaculata* urediniospore germination, germ tubes showing oriented growth, appressoria formation on stomatal apertures and infection foci were measured on six *Brachypodium* accessions **(a)**. One way Analysis of variance (ANOVA) indicated differences among accessions for all parameters (*p*-values 0.0015, 0.0000000008, 0.0000001 and 0.0001 for germination, oriented growth, appressoria and HR, respectively). **(b)** Bd3-1 and Bd30-1 pre-infection structures, including microscopic evaluation of hypersensitive response (HR). Two tailed Student’s *t*-test (*p*-values 0.010, 0.0007, 0.0005 and 0.0002 for germination, oriented growth, appressoria and HR, respectively) indicate significant differences among both accessions for all tested parameters. It is important to note that *P. emaculata* failed to sporulate on any of the accessions tested. For statistical analysis, data from three biological replications was used. Error bars indicate standard deviation from mean.

*P. emaculata* was able to penetrate *Brachypodium* line Bd21, but failed to produce disease symptoms. Therefore, we decided to study genetic variations for NHR by investigating various *Brachypodium* germplasm lines. A total of 38 *Brachypodium* germplasm lines were tested for their response to *P. emaculata* urediniospore inoculation. Out of 38 lines, 32 lines representing different geographical locations in the world with unknown heterozygosity were procured from USDA-GRIN. Six inbred accessions, Bd21, Bd21-3, Bd3-1, Bd18-1, Bd29-1 and Bd30-1, were developed by single-seed descent to increase homozygosity and minimum variations within each line [37]. None of the tested 38 lines showed any disease susceptibility to *P. emaculata* (data not shown). Six *Brachypodium* inbred accessions which were considered to have minimum heterogeneity were further evaluated microscopically for the development of pre-infection structures of *P. emaculata* upon inoculation (Figure 4a). Microscopic evaluations were conducted on 3–4 weeks old plants by studying at least 10 leaves per genotype pooled from three individual plants to represent one biological replication. For each replication, minimum of 100 interaction sites were scored. One way Analysis Of Variance (ANOVA) indicated significant differences among accessions for all tested parameters. Our results indicated a germination rate of more than 80% of *P. emaculata* urediniospores on the leaf surface of each tested inbred accession (Figure 4a). Oriented growth of urediniospore germ tubes was lowest in Bd3-1 (27.8%) and highest in Bd29-1 (84.6%), followed by Bd8-1 (69.7) and Bd30-1 (69.3%) (Figure 4a). Appressoria formation was also highest in Bd30-1 (47.4%) (Figure 4a). Surprisingly, no infection foci were noticed in Bd3-1, whereas infection foci were formed in all other tested inbred accessions (Figure 4a). Infection foci are the sites of infection in mesophyll cells after the fungus penetrates via stomata. Appressoria formation and formation of infection foci are the important steps before colonization of the fungus and/or to trigger elicitor-induced defense response. These results suggest that Bd3-1 is more resistant to *P. emaculata* compared to other inbred accessions tested, while Bd30-1 was somewhat less resistant to *P. emaculata*. Based on the variation in appressoria and infection foci formation, Bd3-1 and Bd30-1 were further evaluated for more comprehensive analyses of these variations (Figure 4b). Data was collected from four separate experiments with eight replications each. Because rust infection foci were not detected in Bd3-1 in previous analyses, both Bd3-1 and Bd30-1 accessions were tested for the presence of hypersensitive cell death at the site of penetration using a light microscope. The percentage of germinating spores showing oriented growth, appressoria formation and infection sites with hypersensitive cell death was higher in Bd30-1 than in Bd3-1 (Figure 4b). Overall, our results indicated an enhanced resistance response in Bd3-1 against *P. emaculata* compared to Bd30-1.

### Transcript profiling of defense-related genes in *P. emaculata*-inoculated Bd21

**Figure 5:**
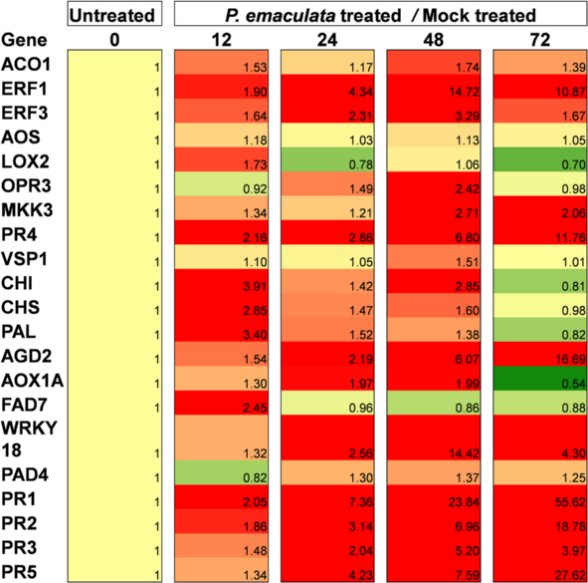
Heat map of transcript profile of defense-related genes in *P. emaculata* inoculated Bd21 leaves. Transcript profiling of defense-related genes in Bd21 leaf tissue collected at 0, 12, 24, 48 and 72 hours after inoculation (hai) with *P. emaculata* or after treatment with water containing 0.05% Tween20 (mock). For each treatment, leaves of at least five plants were pooled for each replication with a total of three replications per treatment. Relative quantification (in fold change) of *P. emaculata* and mock treated samples was calculated in relation to 0 hai and given a value of one. For each time-point, change in gene expression was calculated by measuring ratios of fold change between *P. emaculata* inoculated and mock inoculated samples. Intensity of red and green color indicates the extent of upregulation and downregulation, respectively, with respect to the gene expression at 0 hai which has been normalized to 1 (yellow). Numerical values of fold change are given in parenthesis in each box. 0, 12, 24, 48 and 72 represent hai. Three technical replicates were used for each sample.

To identify the role of known defense genes against switchgrass rust, transcript profiles of 21 representative genes involved in various plant defense pathways were studied. A quantitative real-time PCR (qRT-PCR) method was used to measure transcript level changes of defense-related genes in Bd21 at 0 h (hours), 12 h, 24 h, 48 h and 72 h after *P. emaculata* inoculations (Figure 5). Transcripts of mock inoculated samples collected at 12 h, 24 h, 48 h and 72 h was used as control and the fold change ratio between *P. emaculata* inoculated vs mock was used for analysis. Transcripts of most genes involved in Ethylene (ET), Salicylic Acid (SA) and Jasmonic Acid (JA) biosynthesis or signaling pathways were induced at different time-points after pathogen inoculation depending upon their role in plant defense. For example, transcription factors that play a role in the ethylene signaling pathway, *ERF1* (Ethylene Response Factor 1) and *ERF3* (Ethylene Response Factor 3), were induced more than two folds in first 24 hours after inoculation (hai) and later maintained till 72 hai with slight reduction in expression (Figure 5). Transcript level of *ACO1* (1-aminocyclopropane-1-carboxylic acid oxidase) was also slightly induced with similar pattern to *ERF1* and *ERF3. ACO1* is an ethylene biosynthetic pathway gene and is up-regulated in response to pathogen infection [38]. Among genes encoding enzymes involved in JA biosynthesis such as *LOX2* (Lipoxygenase 2), *OPR3* (12-oxophytodienoate reductase 3) and *AOS* (Allene Oxide Synthase), only *OPR3* was induced by up to two folds at 48 hai (Figure 5). Another gene, *MKK3* which is involved in JA signaling also showed similar trend as *OPR3*. Interestingly, *VSP1* (vegetative storage protein 1), a gene known to be induced by JA, was not induced appreciably at any tested time point compared to mock inoculations. Among SA signaling pathway genes, transcripts of *AGD2* (Aberrant Growth Defects 2) and *AOX1a* (Alternative Oxidase) were highly induced by 16.7 fold at 72 hai and ~2 fold at 48 hai, respectively. Strikingly, all tested pathogenesis-related proteins encoding genes such as *PR1, PR2, PR3, PR4* and *PR5* were significantly induced in response to *P. emaculata* with upward trend in transcript abundance until 72 hai (Figure 5). Among these, *PR1* was the highly induced gene with 55 fold increase at 72 hai (Figure 5). No significant differences in transcript level changes of *FAD7* (omega-3 Fatty Acid Desaturase) and *PAD4* (PhytoAlexin Deficient 4) were observed at tested time-points except *FAD7* was induced by two fold at 12 hai. *WRKY18* was induced up to two fold at 24 hai with highest induction of 14 fold at 72 hai (Figure 5). Genes involved in secondary metabolism, especially during the early steps of phenylpropanoid pathway, such as *CHI* (Chalcone Isomerase), *CHS* (Chalcone Synthase) and *PAL* (Phenylalanine Ammonia-Lyase) were induced by two fold mainly at 12 hai but the induction was below two fold in later time points (Figure 5). Overall, our data suggest that transcripts of many tested plant defense-related genes were induced in Bd21 leaves at various time-points after *P. emaculata* inoculation.

### Basal transcript profiles of plant defense-related genes in Bd3-1, Bd21 and Bd30-1

**Figure 6:**
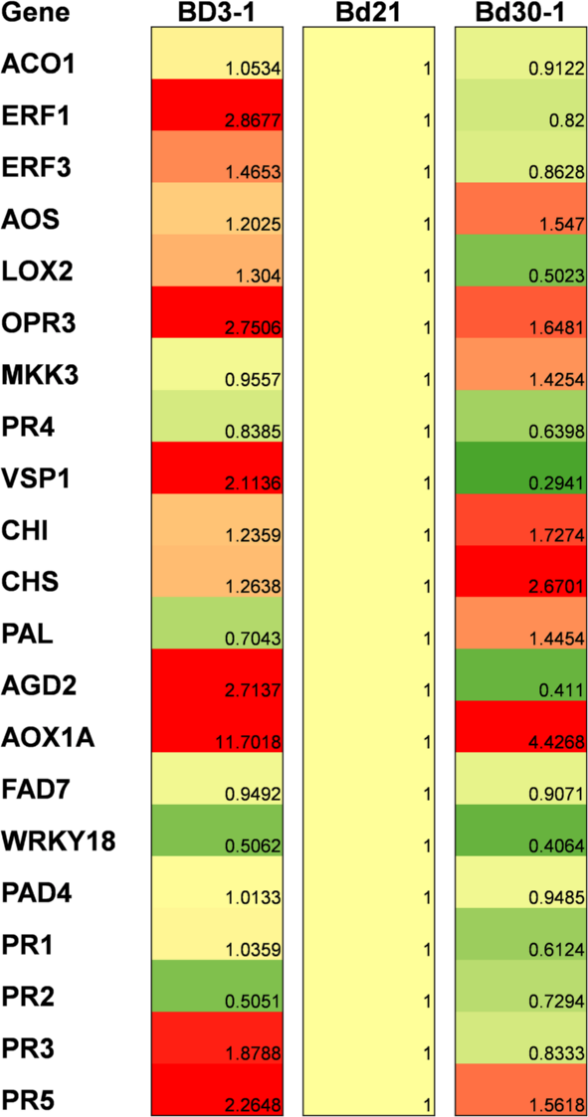
Heat map of basal transcript profile of defense-related genes among Bd3-1, Bd21 and Bd30-1. Transcript profile of defense-related genes was estimated from leaves of Bd3-1, Bd21 and Bd30-1. For each sample, leaves of at least five plants were pooled with three biological replicates per sample. Relative transcript levels were calculated in Bd3-1 and Bd30-1 by keeping transcript levels of Bd21 constant. Intensity of red and green color indicate extent of upregulation and downregulation, respectively, with respect to the gene expression of Bd21 normalized to 1 (yellow). Numerical values of fold change are given in parenthesis in each box. Three technical replicates were used for each sample.

Transcript profiling of Bd21 upon *P. emaculata* inoculation showed induction of most defense-related genes indicating their potential involvement in NHR against *P. emaculata*. In order to explain the variations in resistance in Bd3-1 and Bd30-1, we extended our analysis by conducting a basal transcript profiling of tested defense-related genes in Bd3-1 and Bd30-1 in relation to Bd21. It was surprising to see that some of the genes which were induced after *P. emaculata* inoculations in Bd21 had inherently high basal transcript levels in Bd3-1 which also have more penetration resistance (Figure 6). Transcript levels of *ERF1*, *OPR3*, *VSP1*, *AGD2*, *AOX1A* and *PR5* were more than two folds in Bd3-1 compared to Bd21, whereas, transcript levels of rest of the tested genes in Bd3-1 were either less than two fold or comparable to Bd21 (Figure 6). On the other hand, Bd30-1 did not show higher transcript abundance compared to Bd21 for most of the tested genes except *CHS* and *AOX1A* which showed more than two fold increase (Figure 6). Interestingly, some of the genes such as *VSP1* and *AGD2*, which had higher transcript abundance in Bd3-1, showed two fold decrease in transcript abundance in Bd30-1 (Figure 6). These results correspond with our phenotypic evaluation in which Bd3-1 was inherently more resistant to *P. emaculata* infection when compared to Bd21 and Bd30-1.

## Discussion

Switchgrass rust, caused by *P. emaculata*, is an important disease of switchgrass, but not much attention has been given to this disease so far. Although variations in host resistance have been reported among switchgrass germplasm, those variations have not been exploited so far in switchgrass breeding [8]. In an effort to identify sources of NHR against switchgrass rust, we tested six different monocot plant species belonging to the BEP and PACCMAD clades for their response to *P. emaculata* inoculation (Additional file 5). From this analysis, we determined that *Brachypodium*, where the *P. emaculata* can successfully penetrate, is a suitable model to study nonhost interactions and to identify novel sources of resistance against *P. emaculata*. The inability of *P. emaculata* to successfully penetrate other monocot species tested, such as foxtail millet, corn, sorghum and barley, could be due to the absence of biochemical, topographical and thigmotropic signals from these nonhost species (Figure 2). These signals provide important cues to germinating spores for successful penetration by recognizing the host surface [39]. Based on our results we can speculate that these signals were recognized by germinating urediniospores on leaf surfaces of rice and *Brachypodium* to form appressoria (Figures 2e, f and 3). In some instances, intercellular fungal hyphal growth was also observed between mesophyll cells, but the presence of haustoria was not confirmed in *Brachypodium* (Figure 3f). Occasionally, *Brachypodium* also exhibited HR-related cell death at the site of rust infection which is only visible under the microscope (Figure 3d). Similar observations were also reported earlier for *Puccinia* species which are pathogenic to wheat, and the growth of pre-infection structures was far greater on *Brachypodium* than on rice [24]. Additionally, so far none of the rust pathogens are able to breach NHR imparted by rice [22]. Wheat stripe rust fungus *P. striiformis* and wheat stem rust fungus *P. graminis* f. sp. *tritici* showed successful colonization and sporulating pustules on some of the *Brachypodium* accessions [24,33]. Interestingly, in our study, *P. emaculata* failed to produce successful disease establishment in the form of rust pustules on the *Brachypodium* accessions tested. The failure of *P. emaculata* to colonize on other cereal species can be better explained by studying the evolutionary relationship among different species of genus *Puccinia*. An internal transcribed spacer (ITS) primer-based phylogenetic tree placed *P. emaculata* closer to *P. asparagi*, an asparagus rust pathogen, than other cereal rusts [8]. Detailed phylogenetic information is needed to study the evolution of *P. emaculata* and to identify the precise relationship of *P. emaculata* with other rust fungi to explain variability in host range.

Variations in pre-infection structures among different *Brachypodium* accessions can be classified under type I NHR (Figure 4). However, in later stages, intercellular growth of fungal hyphae in mesophyll cells followed by hypersensitive response is observed in all tested *Brachypodium* accessions also indicating a strong apoplastic defense response. Among tested accessions, Bd3-1 and Bd30-1 were most resistant and least resistant to switchgrass rust, respectively (Figure 4). Similar to our results, it has been previously reported that Bd3-1 was more resistant than Bd30-1 against *P. graminis* f. sp. *lolii*, *phlei-pratensis* and *tritici* [34]. A genetic mapping population followed by QTL analyses can be used in future to capture genetic variation among these accessions.

To understand the role of known defense genes in NHR of *Brachypodium* against switchgrass rust, we conducted a transcript profiling of defense-related genes in Bd21 leaves at various time-points after *P. emaculata* inoculations (Figure 5). Several genes involved in JA, ET and SA pathways were induced in *P. emaculata-*inoculated leaves of Bd21 (Figure 5). JA and ET pathways are usually involved in defense against necrotrophic pathogens, whereas the SA pathway is involved in defense against biotrophic pathogens [40]. In Arabidopsis, *AOX1a* expression is controlled by H_2_O_2_ signaling, SA application and a pathway involving *EDS1* and *PAD4* [41]. AOX acts as an antioxidant to combat excessive reactive oxygen species production during HR response during defense against pathogens [42]. *AOX1a* was also induced at 48 to 72 h after rust inoculation in Bd21 (Figure 5) indicating its potential involvement in post-penetration resistance which is often associated with HR response. Another protein, AGD2, which is involved in lysine biosynthesis, acts as a negative regulator of plant defense against biotrophic pathogens [43,44]. *AGD2* was induced up to 16 fold in Bd21 after pathogen inoculation (Figure 5). Ethylene response factors encoding genes, *ERF1* and *ERF2* were also induced after rust inoculation which was surprising because these genes are often involved in resistance against necrotrophic pathogens. Role of ethylene response factors has been reported previously in *Medicago truncatula* for resistance against *R. solani* [45]. Similarly, a JA biosynthetic pathway gene, *OPR3* was also induced in Bd21 in response to rust inoculation (Figure 5). JA biosynthesis and elicitation of JA response upon wounding and stress are associated with increased transcriptional activity of this gene [46]. Contrary to *OPR3*, *VSP1* was not induced after pathogen inoculation (Figure 5). *VSP1* is generally induced by JA, and a mutant with constitutive expression of *VSP1* shows enhanced resistance to pathogens [47,48]. However, in the present context, lack of induction of *VSP1* against biotrophic pathogen makes sense because of its involvement in JA signaling. However, induction of *OPR3* and its role in defense against a biotrophic rust pathogen is difficult to explain. Induction of transcript levels of phenylpropanoid pathway genes (*PAL*, *CHI* and *CHS*) during early phases of infection was also noticed (Figure 5). Phenylpropanoid pathway compounds are generally involved in plant defense by acting as barriers against infection or as signaling molecules [49]. Induced transcripts of all tested *PR* genes with *P. emaculata* inoculation was interesting but not surprising due to perhaps their involvement in plant defense against invading pathogens [50].

To test if the defense-related genes which were induced in Bd21 in response to *P. emaculata* inoculation are also responsible for variations in resistance among *Brachypodium* inbred accessions, we extended our analyses by studying the basal transcript abundance of tested genes in Bd3-1 and Bd30-1 relative to Bd21. Surprisingly, the genes such as *ERF1*, *OPR3*, *AGD2* and *AOX1A* which were induced in response to *P. emaculata* were also showed more than two fold basal transcript abundance in Bd3-1 relative to Bd21 (Figure 6). Coincidently, Bd3-1 was also selected as the most resistance inbred accession among tested accessions. It could be possible that some or all of these genes were involved in high penetration resistance in Bd3-1 against *P. emaculata*. Additionally, out of five *PR* genes which were induced in Bd21 after rust inoculation, only *PR*3 and *PR5* showed two fold transcript abundance in Bd3-1 relative to Bd21 or Bd30-1 (Figure 6). *PR3* encodes endochitinase which could act against fungal pathogens [50]. In Arabidopsis, *PR5* (a thaumatin) gene is induced in response to the SA pathway against biotrophic pathogens [51]. Previously, the transcript level of *PR5* was shown to be associated with incompatible interactions of wheat with wheat stripe rust [52]. Considering these studies, higher transcript levels of *PR3* and *PR5* in Bd3-1 (Figure 6) may be directly correlated with enhanced resistance against switchgrass rust.

## Conclusion

We characterized switchgrass rust interactions with six monocot nonhost species and identified *Brachypodium* as a suitable nonhost model to study switchgrass rust. Analyses of *Brachypodium*-switchgrass rust interactions suggest type I NHR responses exhibited by *Brachypodium*. Genetic variation in resistance was reported among *Brachypodium* accessions against switchgrass rust. Among tested accessions, Bd3-1 exhibited more resistance against *P. emaculata*. These variations were further characterized at the molecular level by studying the transcript profiling of representative defense-related genes after *P. emaculata* inoculations in Bd21 and by measuring the basal transcript levels of these genes in a few accessions. Transcript profiling indicated involvement of various defense pathways in NHR imparted by *Brachypodium* against *P. emaculata*. Overall, the current study provides an avenue to identify novel sources of resistance against switchgrass rust by utilizing the extensive genomic and genetic resources available for *Brachypodium*.

## Methods

### Plant material and growth conditions

*Brachypodium* inbred accessions Bd21, Bd21-3, Bd3-1, Bd18-1, Bd29-1 and Bd30-1 were kindly provided by Dr. David Garvin, ARS-USDA. Thirty-two *Brachypodium* germplasm lines were procured from USDA-GRIN (United States Department of Agriculture-Germplasm Resources Information Network) (Additional file 1). Seeds of selected monocots, corn, sorghum, barley, rice and foxtail millet were procured from Drs. Xin Ding and Malay Saha at The Samuel Roberts Noble Foundation, Ardmore, Oklahoma, USA. Switchgrass cv. Summer, which is susceptible to *P. emaculata*, was used for multiplication of urediniospores [8]. *Brachypodium*, switchgrass and selected monocots belonging to the PACCMAD and BEP clades were planted and grown in a greenhouse with daytime and nighttime temperatures of 22°C and 18°C, respectively. Plant inoculations with rust were conducted in a biosafety level 2 room and kept in growth chambers at 29/22°C day/night temperature, 16 h photoperiod, 90% relative humidity and photon flux density 150–200 μmolm^−2^ s^−1^.

### Switchgrass rust inoculation and screening

Urediniospores of *P. emaculata* were originally collected from switchgrass fields in Ardmore, Oklahoma [8]. Plant material was inoculated with freshly collected urediniospores from controlled inoculations in a growth chamber. Urediniospore suspension at a concentration of 10^5^ spores/ml was prepared in water containing 0.05% Tween-20. Plants were inoculated with spray inoculation using an artist’s airbrush (Paasche Airbrush Company, Chicago, Illinois, USA) followed by misting with distilled water. For mock inoculation, plants were sprayed with water containing 0.05% Tween-20. Both *P. emaculata* and mock inoculated plants were kept at 22°C under dark conditions for 16 hours before putting them back in a growth chamber under the above described environmental conditions. Rust-inoculated plants were screened after two weeks for disease susceptibility. For Bd21 time course experiment, similar inoculation procedure and environmental conditions were followed.

### Microscopic evaluation/screening

Microscopic evaluation of rust urediniospore pre-infection structures was conducted on detached leaves. Minimum of 10 newly emerged leaves for each genotype were clipped from 3–4 week old plants and arranged on wet paper towels in Petri plates to represent one biological replicate. Statistical analyses of microscopic observations was conducted by performing one way ANOVA and Student’s *t*-test. Spray-inoculation of urediniospore suspension was followed as described in the previous section. Sprayed leaves were kept under the same environmental conditions described in the previous section. Microscopic screening experiment was conducted at least three times for consistency. Microscopic evaluations were conducted 72 hours after rust inoculation. For microscopic evaluations, leaves were first immersed in 1X PBS (phosphate-buffered saline) buffer containing 0.05% Tween-20 and 10 μg/ml wheat germ agglutinin conjugated with Alexa-fluor-488 (Invitrogen, Carlsbad, California, USA) for 10 minutes followed by three washings with 1X PBS buffer [16]. Leaf samples were arranged on a glass slide with a cover slip on top and visualized under an Olympus BX41 epifluorescence microscope (Olympus Corporation, Tokyo, Japan) and/or a Leica TCS SP2 AOBS confocal laser scanning microscope (Leica Microsystems CMS GmbH, Mannheim, Germany) with UV excitation. To evaluate percent germination, urediniospores which formed germ tubes >10 μm were considered germinated at 24 hours after inoculation. Germinated spores were evaluated for oriented growth and appressoria formation on top of stomatal opening. More than 100 spores were evaluated from five independent leaves of each genotype for each replication. Scanning electron microscopy was conducted using Hitachi TM3000, a tabletop scanning electron microscope (Hitachi High-Technologies Corporation, Tokyo, Japan) to analyze and score the pre-infection structures of *P. emaculata*.

### DAB (3,3′-diaminobenzidine) staining

For DAB staining, infected leaves were placed in a freshly prepared solution of DAB-HCl (pH 3.8) at a concentration of 1 mg ml^−1^ for eight hours at room temperature. Leaf chlorophylls were removed with 95% ethanol before visualizing under a light microscope as described by Ishiga et al. [53].

### RNA isolation and quantitative real-time PCR

Leaf tissue was collected from 3–4 week old *Brachypodium* plants. Leaves of five plants were pooled for each treatment per replicate and minimum of three biological replicates were used for RNA isolation. Collected leaf samples were used for total RNA isolation by the hot phenol/guanidinium thiocyanate method (TRIzol® Reagent, Invitrogen, Carlsbad, California, USA). First-strand cDNA was synthesized from 2 μg of total RNA using the SuperScript® III First-Strand Synthesis System (Invitrogen, Carlsbad, California, USA). Quantitative real-time PCR (RT-qPCR) analysis was conducted on CFX Connect™ Real-Time PCR Detection System (Bio-Rad, Hercules, California, USA) and Applied Biosystems 7900HT Fast Real-Time PCR system (Life Technologies, Grand Island, New York, USA) by following the manufacturer’s instructions. For RT-qPCR analyses, three biological replicates per treatment and three experimental replicates per sample were used. Data was analyzed by software DataAssist™ v3.01 (Life Technologies, Grand Island, New York, USA) by calibrating to housekeeping control gene, *Ubiquitin*. Relative quantification was measured using 2^-ΔΔCT^ method [54]. PCR efficiencies for each PCR well were calculated by using software, LinRegPCR [55]. Average PCR efficiency for each primer pair is given in Additional file 6. Relative quantification (in fold change) of transcripts was estimated in reference to Bd21 with selected value of one (Additional file 3 and Additional file 4). Detail of selected defense-related genes and their primer sequences is given in Additional file 2.

## Additional files

Additional file 1: Table S1. List of *Brachypodium* accessions procured from United States Department of Agriculture-Germplasm Resources Information Network (USDA-GRIN) and tested for disease reaction against *P. emaculata*. Additional file 2:Table S2.
*Brachypodium* defense-related genes and their primer sequences used for quantitative RT-PCR analysis. Additional file 3: Table S3. Relative quantification (RQ) and P-value of defense related genes in Bd21 leaf tissue at 0, 12, 24, 48 and 72 hours after *P.emaculata* inoculation. Additional file 4: Table S4. Relative quantification (RQ) and P-value of basal transcript level of defense related genes in Bd3-1 and Bd30-1 relative to Bd21. Additional file 5:
**Taxonomy tree of tested monocot species.** Seven monocot species, *Zea mays*, *Sorghum bicolor*, *Panicum virgatum*, *Setaria italica*, *Oryza sativa*, *Brachypodium distachyon* and *Hordeum vulgare*, were tested for nonhost/host resistance against switchgrass rust pathogen *P. emaculata*. Taxonomic information is based on the NCBI (National Center for Biotechnology Information) database. Additional file 6: Table S5. Average PCR efficiencies and correlation coefficient of each primer pair used for qRT-PCR. Data points with PCR efficiencies of more than 1.8 were used for analysis.

## Abbreviations

DOE: Department of energy
NHR: Nonhost resistance
HR: Hypersensitive response
QTL: Quantitative trait loci
BSMV: Barley stripe mosaic virus
ET: Ethylene
JA: Jasmonic acid
SA: Salicylic acid
