# Supplementary material for: Characterization of Brachypodium distachyon as a nonhost model against switchgrass rust pathogen Puccinia emaculata
Source: BMC Plant Biol. 2015 May 8;15:113. doi: 10.1186/s12870-015-0502-9 (PMC4424542; doi:10.1186/s12870-015-0502-9)
Supplement: Additional file 1: Table S1. — List of Brachypodium accessions procured from United States Department of Agriculture-Germplasm Resources Information Network (USDA-GRIN) and tested for disease reaction against P. emaculata. [file 12870_2015_502_MOESM1_ESM.docx]

**Supplementary Table 1.** List of Brachypodium accessions procured from United States Department of Agriculture-Germplasm Resources Information Network (USDA-GRIN) and tested for disease reaction against *P. emaculata*.

| **GRIN Accession number** | **Plant species** |
| --- | --- |
| PI208216 | Brachypodium distachyon |
| PI219961 | Brachypodium distachyon |
| PI219965 | Brachypodium distachyon |
| PI219968 | Brachypodium distachyon |
| PI219971 | Brachypodium distachyon |
| PI220567 | Brachypodium distachyon |
| PI-226629 | Brachypodium distachyon |
| PI-227011 | Brachypodium distachyon |
| PI-239713 | Brachypodium distachyon |
| PI-239715 | Brachypodium distachyon |
| PI-239716 | Brachypodium distachyon |
| PI-250647 | Brachypodium distachyon |
| PI-253334 | Brachypodium distachyon |
| PI-254867 | Brachypodium distachyon |
| PI-254868 | Brachypodium distachyon |
| PI-287783 | Brachypodium distachyon |
| PI-317418 | Brachypodium distachyon |
| PI-321403 | Brachypodium distachyon |
| PI-372187 | Brachypodium distachyon |
| PI-422452 | Brachypodium distachyon |
| PI-533015 | Brachypodium distachyon |
| PI-639818 | Brachypodium distachyon |
| W6-19177 | Brachypodium distachyon |
| W6-21792 | Brachypodium distachyon |
| W6-39234 | Brachypodium distachyon |
| W6-39257 | Brachypodium distachyon |
| W6-39266 | Brachypodium distachyon |
| W6-39272 | Brachypodium distachyon |
| W6-39278 | Brachypodium distachyon |
| W6-39285 | Brachypodium distachyon |
| W6-39289 | Brachypodium distachyon |
| W6-39301 | Brachypodium distachyon |
