# Supplementary material for: Characterization of Brachypodium distachyon as a nonhost model against switchgrass rust pathogen Puccinia emaculata
Source: BMC Plant Biol. 2015 May 8;15:113. doi: 10.1186/s12870-015-0502-9 (PMC4424542; doi:10.1186/s12870-015-0502-9)
Supplement: Additional file 2:Table S2. — Brachypodium defense-related genes and their primer sequences used for quantitative RT-PCR analysis. [file 12870_2015_502_MOESM2_ESM.docx]

**Supplementary table 2.** *Brachypodium* defense related genes and their primer sequences used for quantitative RT-PCR analysis.

| **Gene** | **Primer name** | **Sequence (5´→3´)** |
| --- | --- | --- |
| *ACO1* | ACO1_F | CATATTCCATCAGGGGAGAAGC |
|  | ACO1_R | CTTCCACTGCCATACTCAGCAC |
| *AGD2* | AGD2_F | GTACCCAGAAGCGAAGGTCATC |
|  | AGD2_R | TAGCCTTGGTAGCCTTCAGGAG |
| *CHI* | Bd_CHI_R | TGGAAAGTGGAAGGTGACGAC |
|  | CHI_F | GAGATCAAGGGGTCACAGTACG |
| *CHS* | CHS_F | CTGACGTTCCATCTCCTCAAG |
|  | CHS_R | CCGCTCCTTGTTCAAGTTAATC |
| *FAD7* | FAD7_F | CGTGGAAAGGAATGGAGCTATC |
|  | FAD7_R | CCCTGTAGTATTTCCCCAGCAC |
| *MKK3* | MKK3_F | TCTGCTGATATCTGGAGCCTTG |
|  | MKK3_R | TACACCCGGTCCCTCATATCTC |
| *PAL* | PAL_F | ATTCAGGCTATCCTTGCTGAGG |
|  | PAL_R | AGGAGCTTCCTTCCAAGATGTG |
| *PR2* | PR2_F | CATCAACTCCATGCGGATCTAC |
|  | PR2_R | GGCGATGTACTTGATGTTGACC |
| *PR4* | PR4_F | CTGGACCTGGACTGGGACAC |
|  | PR4_R | TTATCACCACAGTCGACGAACTC |
| *VSP1* | VSP1_F | GTTCAAGACCTTCCTGCTCACC |
|  | VSP1_R | AGCTGCTGCCCTCAATATAAGC |
| *WRKY18* | WRKY18_F | GCTTAGAGACGACGGCACTTAC |
|  | WRKY18_R | TTGATACCCATCCTTCACAACG |
| **AOS* | AOS_F | AACAATGGTGCGATCGGCTTCG |
|  | AOS_R | CCGTGGGACCTCAAGGTAAAGTTC |
| **AOX1A* | AOX1A_F | ACTACGCCTCGGACATCCATTAC |
|  | AOX1A_R | AGGCATCGACCGTCCATTTGAG |
| **ERF-1* | ERF-1_F | TGGTGCCGTGTGAAATTTGTCG |
|  | ERF-1_R | CAGATTTCGCTGCACCACTTGC |
| **ERF3* | ERF3_F | AGGATTTGCGCCAAATTGTGCAG |
|  | ERF3_R | ATCCAAGAGGGCGTGCAAAGAG |
| **LOX2* | LOX2_F | GCGGCGTTCGAGAAGTTCAATG |
|  | LOX2_R | GTCCTGGTTATTGTTTCGCTCGTC |
| **OPR3* | OPR3_F | ACCCATTTCTTCTCGAATGATCCC |
|  | OPR3_R | ACACGTGCAAGTACGGAAAGAAAG |
| **PAD4* | PAD4_F | GCGAGAGAAGGTATGAGCTGTTTG |
|  | PAD4_R | TGGTGCAGCCAGTAAAGGTTCC |
| **PR-1* | PR-1_F | AGCTCTGGCATCATCAGCATCC |
|  | PR-1_R | CGTTGTGTGGGTCCAGGAAATC |
| **PR-3* | PR-3_F | GCTCGGCTGATTGTTCAACACG |
|  | PR-3_R | TTGCCCGAACCACAAATATGCC |
| **PR-5* | PR-5_F | CCGACCGATTACTCCAGGTTCTTC |
|  | PR-5_R | TAATTAGCTCGCTCGCTCGCTTG |
| *Ubiquitin* | UBQ_F | TCCACACTCCACTTGGTGCT |
|  | UBQ_R | GAGGGTGGACTCCTTTTGGA |

*Primer sequence information was received from Mandadi and Scholthof (2012).

**References:**

# Mandadi, K.K. and Scholthof, K.B.G. (2012) Characterization of a viral synergism in the monocot Brachypodium distachyon reveals distinctly altered host molecular processes associated with disease. *Plant Physiol*. 160, 1432-1452.
