# Supplementary figures and images for: Characterization of Brachypodium distachyon as a nonhost model against switchgrass rust pathogen Puccinia emaculata
Source: BMC Plant Biol. 2015 May 8;15:113. doi: 10.1186/s12870-015-0502-9 (PMC4424542; doi:10.1186/s12870-015-0502-9)

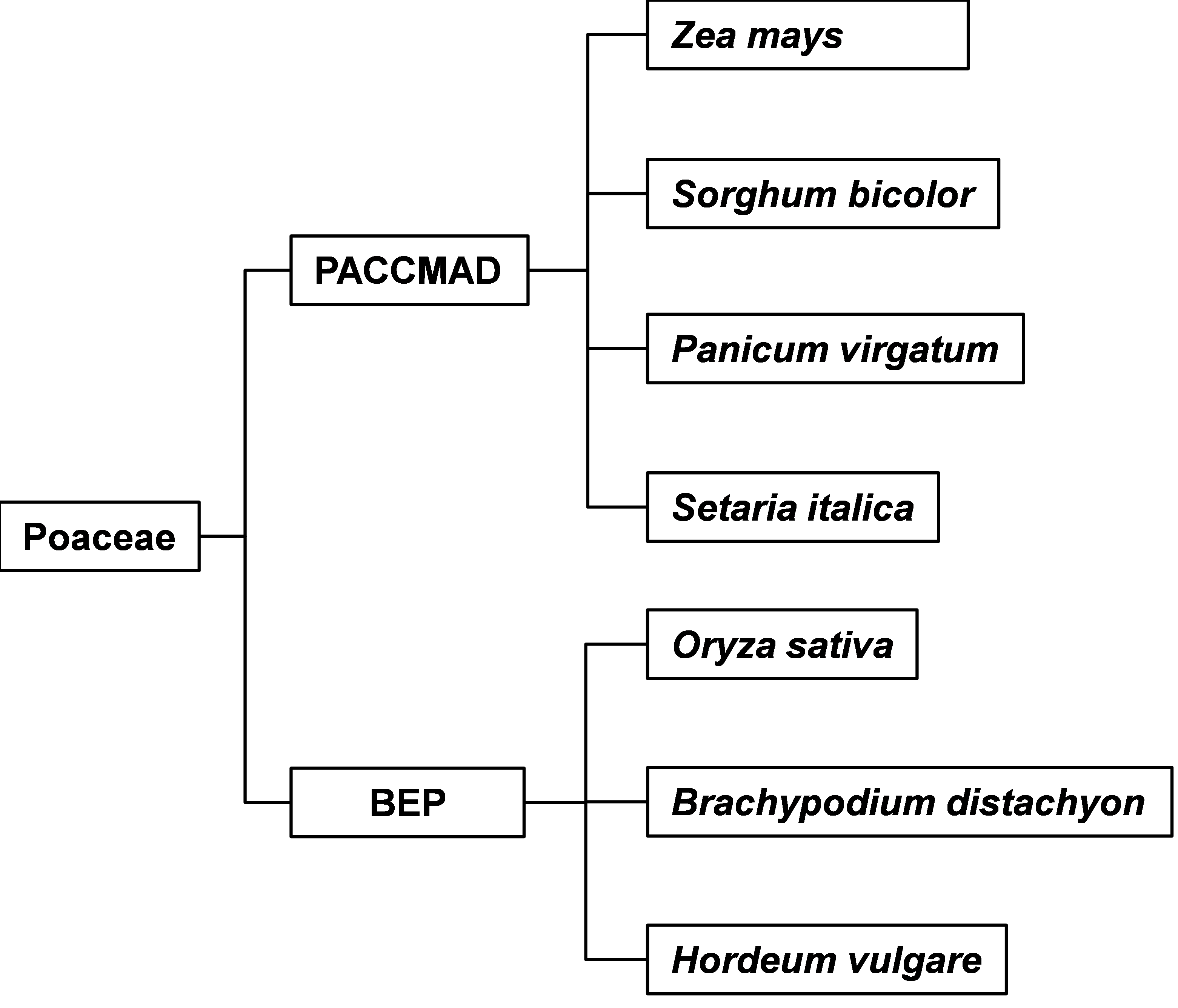

Supplement: Additional file 5: — Taxonomy tree of tested monocot species. Seven monocot species, Zea mays, Sorghum bicolor, Panicum virgatum, Setaria italica, Oryza sativa, Brachypodium distachyon and Hordeum vulgare, were tested for nonhost/host resistance against switchgrass rust pathogen P. emaculata. Taxonomic information is based on the NCBI (National Center for Biotechnology Information) database. [file 12870_2015_502_MOESM5_ESM.tiff]
