# Supplementary material for: Characterization of Brachypodium distachyon as a nonhost model against switchgrass rust pathogen Puccinia emaculata
Source: BMC Plant Biol. 2015 May 8;15:113. doi: 10.1186/s12870-015-0502-9 (PMC4424542; doi:10.1186/s12870-015-0502-9)
Supplement: Additional file 6: Table S5. — Average PCR efficiencies and correlation coefficient of each primer pair used for qRT-PCR. Data points with PCR efficiencies of more than 1.8 were used for analysis. [file 12870_2015_502_MOESM6_ESM.docx]

**Supplementary table 5.** Average PCR efficiencies and correlation coefficient of each primer pair used for qRT-PCR. Data points with PCR efficiencies of more than 1.8 were used for analysis.

| **Gene** | **Primer Pair** | **Average PCR efficiency** | **Correlation coefficient (R^2^)** |
| --- | --- | --- | --- |
| *ACO1* | ACO1_F & ACO1_R | 1.961 | 1.000 |
| *AGD2* | AGD2_F & AGD2_R | 1.949 | 1.000 |
| *CHI* | CHI_R & CHI_F | 1.959 | 1.000 |
| *CHS* | CHS_F & CHS_R | 1.979 | 1.000 |
| *FAD7* | FAD7_F & FAD7_R | 1.960 | 1.000 |
| *MKK3* | MKK3_F & MKK3_R | 1.947 | 1.000 |
| *PAL* | PAL_F & PAL_R | 1.940 | 1.000 |
| *PR2* | PR2_F & PR2_R | 1.927 | 0.999 |
| *PR4* | PR4_F & PR4_R | 1.946 | 1.000 |
| *VSP1* | VSP1_F & VSP1_R | 1.931 | 1.000 |
| *WRKY18* | WRKY18_F & WRKY18_R | 1.972 | 1.000 |
| *AOS* | AOS_F & AOS_R | 1.949 | 0.997 |
| *AOX1A* | AOX1A_F & AOX1A_R | 1.970 | 1.000 |
| *ERF-1* | ERF-1_F & ERF-1_R | 1.963 | 1.000 |
| *ERF3* | ERF3_F & ERF3_R | 1.964 | 0.999 |
| *LOX2* | LOX2_F & LOX2_R | 1.983 | 1.000 |
| *OPR3* | OPR3_F & OPR3_R | 1.949 | 1.000 |
| *PAD4* | PAD4_F & PAD4_R | 1.978 | 1.000 |
| *PR-1* | PR-1_F & PR-1_R | 1.990 | 0.998 |
| *PR-3* | PR-3_F & PR-3_R | 2.000 | 1.000 |
| *PR-5* | PR-5_F & PR-5_R | 1.962 | 1.000 |
| *Ubiquitin* | UBQ_F & UBQ_R | 1.973 | 1.000 |
